# Mitochondrial and Plasma Membrane Pools of Stomatin-Like Protein 2 Coalesce at the Immunological Synapse during T Cell Activation

**DOI:** 10.1371/journal.pone.0037144

**Published:** 2012-05-18

**Authors:** Darah A. Christie, Mark G. Kirchhof, Santosh Vardhana, Michael L. Dustin, Joaquín Madrenas

**Affiliations:** 1 The Centre for Human Immunology, Robarts Research Institute, and the Departments of Microbiology and Immunology, and Medicine, The University of Western Ontario, London, Ontario, Canada; 2 Program in Molecular Pathogenesis, Skirball Institute of Biomolecular Medicine, New York, New York, United States of America; 3 Department of Microbiology and Immunology, McGill University, Montreal, Quebec, Canada; University of Montreal, Canada

## Abstract

Stomatin-like protein 2 (SLP-2) is a member of the stomatin – prohibitin – flotillin – HflC/K (SPFH) superfamily. Recent evidence indicates that SLP-2 is involved in the organization of cardiolipin-enriched microdomains in mitochondrial membranes and the regulation of mitochondrial biogenesis and function. In T cells, this role translates into enhanced T cell activation. Although the major pool of SLP-2 is associated with mitochondria, we show here that there is an additional pool of SLP-2 associated with the plasma membrane of T cells. Both plasma membrane-associated and mitochondria-associated pools of SLP-2 coalesce at the immunological synapse (IS) upon T cell activation. SLP-2 is not required for formation of IS nor for the re-localization of mitochondria to the IS because SLP-2-deficient T cells showed normal re-localization of these organelles in response to T cell activation. Interestingly, upon T cell activation, we found the surface pool of SLP-2 mostly excluded from the central supramolecular activation complex, and enriched in the peripheral area of the IS where signalling TCR microclusters are located. Based on these results, we propose that SLP-2 facilitates the compartmentalization not only of mitochondrial membranes but also of the plasma membrane into functional microdomains. In this latter location, SLP-2 may facilitate the optimal assembly of TCR signalosome components. Our data also suggest that there may be a net exchange of membrane material between mitochondria and plasma membrane, explaining the presence of some mitochondrial proteins in the plasma membrane.

## Introduction

Stomatin-like protein 2 (SLP-2), a widely expressed mitochondrial protein identified in proteome analyses from several tissues and species, is a member of the stomatin family as well as the stomatin – prohibitin – flotillin – HflC/K (SPFH) superfamily [1], [2], [3], [4]. In mitochondria, SLP-2 associates tightly with the mitochondrial inner membrane. How this association occurs is unclear because, unlike other stomatin family members, SLP-2 lacks a putative transmembrane domain. It contains though a SPFH domain, a highly conserved region found in SPFH superfamily members [5], [6], [7], [8], and this domain has been linked to protein: lipid membrane interactions [9], [10], [11].

Recent studies have begun to shed light on the function of SLP-2. Similar to other SPFH family members, SLP-2 has been found in large protein complexes and has been shown to interact with mitochondrial proteins, including prohibitin (PHB) 1 and 2 as well as mitofusin 2 [5], [6], [8]. We also have shown that SLP-2 binds directly to cardiolipin and can regulate the localization of the prohibitin complex to cardiolipin-enriched domains in the mitochondrial membrane [12]. Furthermore, over-expression of SLP-2 leads to increased mitochondrial biogenesis and function [5], [6], [8] whereas SLP-2 depletion results in decreased mitochondrial transmembrane potential, reduced mitochondrial calcium uptake in response to cell stimulation, enhanced apoptotic responses to cell stress and increased degradation of mitochondrial proteins [5], [8], [12], [13], [14] (Christie et al, manuscript submitted). All this evidence led us to propose that SLP-2 regulates all these mitochondrial functions (i.e. energy production, calcium buffering and apoptosis) by organizing mitochondrial membranes into defined cardiolipin-enriched microdomains, which then facilitate the optimal assembly of membrane-associated molecular complexes.

Although most recent work on SLP-2 has focused on the function in mitochondria, we originally identified SLP-2 in the glycolipid-enriched, detergent-insoluble microdomains of human T cells activated through the T cell receptor [12]. In these cells, we demonstrated that SLP-2 was recruited to glycolipid-enriched detergent-insoluble microdomains and interacted with numerous components of the TCR signalosome upon T cell stimulation, including CD3, ZAP-70, LAT, lck and PLCγ. Furthermore, the level of SLP-2 expression correlated with the response of T cells to stimulation. Under conditions of SLP-2 over-expression, T cells had significantly increased responses while knockdown of SLP-2 resulted in significantly decreased T cell signalling. Together, these results suggested that SLP-2 plays an important role in T cell activation, either by increasing mitochondrial biogenesis [6] or by regulating T cell signalling at the plasma membrane [12].

To investigate the role of SLP-2 during T cell activation, we utilized a Jurkat T cell line stably transfected with an inducible construct encoding either full-length SLP-2 tagged with gfp or a gfp-tagged mutant construct lacking the amino-terminal region. As expected, we found that most SLP-2 was associated with mitochondria. This primary location was determined by an amino-terminal mitochondrial-targeting sequence of SLP-2. Interestingly, a small pool of SLP-2 was also detected in association with the plasma membrane. Upon T cell stimulation both pools of SLP-2 coalesced at the immunological synapse (IS), and localized mostly in the peripheral supramolecular activation complex (pSMAC). SLP-2 was not required for mitochondrial trafficking to the IS. Our data indicate that there is a small pool of SLP-2 in the plasma membrane that, by analogy with the mitochondrial pool of this protein, may play a role in the compartmentalization of the plasma membrane, ensuring optimal assembly of multimolecular complexes.

## Results

### The Largest Cellular Pool of SLP-2 is Associated with Mitochondria

**Figure 1:**
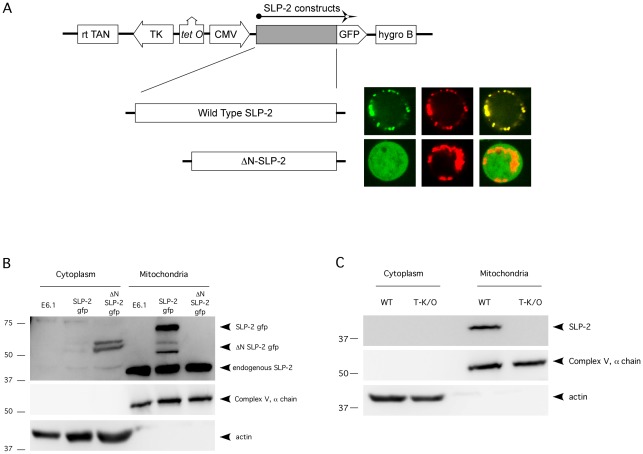
SLP-2 is a mitochondrial protein. A) Full-length SLP-2-gfp and ΔN-SLP-2-gfp were subcloned into a doxycycline-inducible vector and transfected into Jurkat T cells. These stable transfectants were imaged by confocal microscopy for SLP-2-gfp (first column of micrographs – green), or after staining with MitoTracker Red (second column of micrographs – red). The third colum of photomicrographs show overlapping of red and green signals as yellow signal. B) Mitochondrial and cytosolic fractions of parental, SLP-2-gfp and ΔN-SLP-2-gfp T cell transfectants were isolated by differential centrifugation and immunoblotted for SLP-2, the α-subunit of ATP synthase and actin. C) Mitochondrial and cytosolic fractions were isolated from T cells of wild type mice and T-cell specific SLP-2 conditional knockout mice and blotted as in B. These results are representative of at least 3 independent experiments, and of more than 100 imaged cells.

In order to assess the subcellular localization and movement of SLP-2 in T cells, we constructed stably transfected Jurkat T cells with a doxycycline inducible construct encoding SLP-2 tagged with gfp at the C-terminus. In addition, we also constructed a truncated version of SLP-2 lacking the N-terminus of the protein (ΔN-SLP-2-gfp), as this region contains a predicted mitochondrial targeting sequence [8]. Transfected T cells were visualized using confocal microscopy (figure 1A). As expected, we found that most SLP-2-gfp was distributed in aggregates within the cytosol, which were shown to be mitochondria by staining with the mitochondrial dye MitoTracker Red. In the absence of the predicted mitochondrial targeting sequence (ΔN-SLP-2-gfp), the protein failed to partition selectively in mitochondria and was instead found in a diffuse cytosolic pattern, confirming the functionality of the mitochondrial-targeting domain in the N-terminus of SLP-2.

The mitochondrial localization of SLP-2 was further supported by subcellular fractionation and western blotting. As shown in figure 1B, both endogenous SLP-2 and SLP-2-gfp were detected in the mitochondrial but not the cytosolic fractions of transfected Jurkat T cells. The SLP-2-gfp blots showed intermediate degradation products from the full length chimeric construct. The ΔN-SLP-2-gfp was absent in the mitochondrial fraction and detected only in the cytosolic fraction, further confirming the presence of a mitochondrial targeting domain at the amino terminus of SLP-2.

The previous results were corroborated using T cells from wildtype and T-cell-specific SLP-2 knockout mice (figure 1C). Similar to human SLP-2, murine SLP-2 also partitioned in mitochondria. As expected, the SLP-2 T-K/O cells had no detectable SLP-2, verifying the identity of the mitochondrial SLP-2 band in the wild type mice. Altogether, these results confirmed the predominant mitochondrial location of SLP-2 and validated the use of the inducible SLP-2-gfp transfected T cells.

### Detection of a Small Pool of SLP-2 Associated with the Plasma Membrane

**Figure 2:**
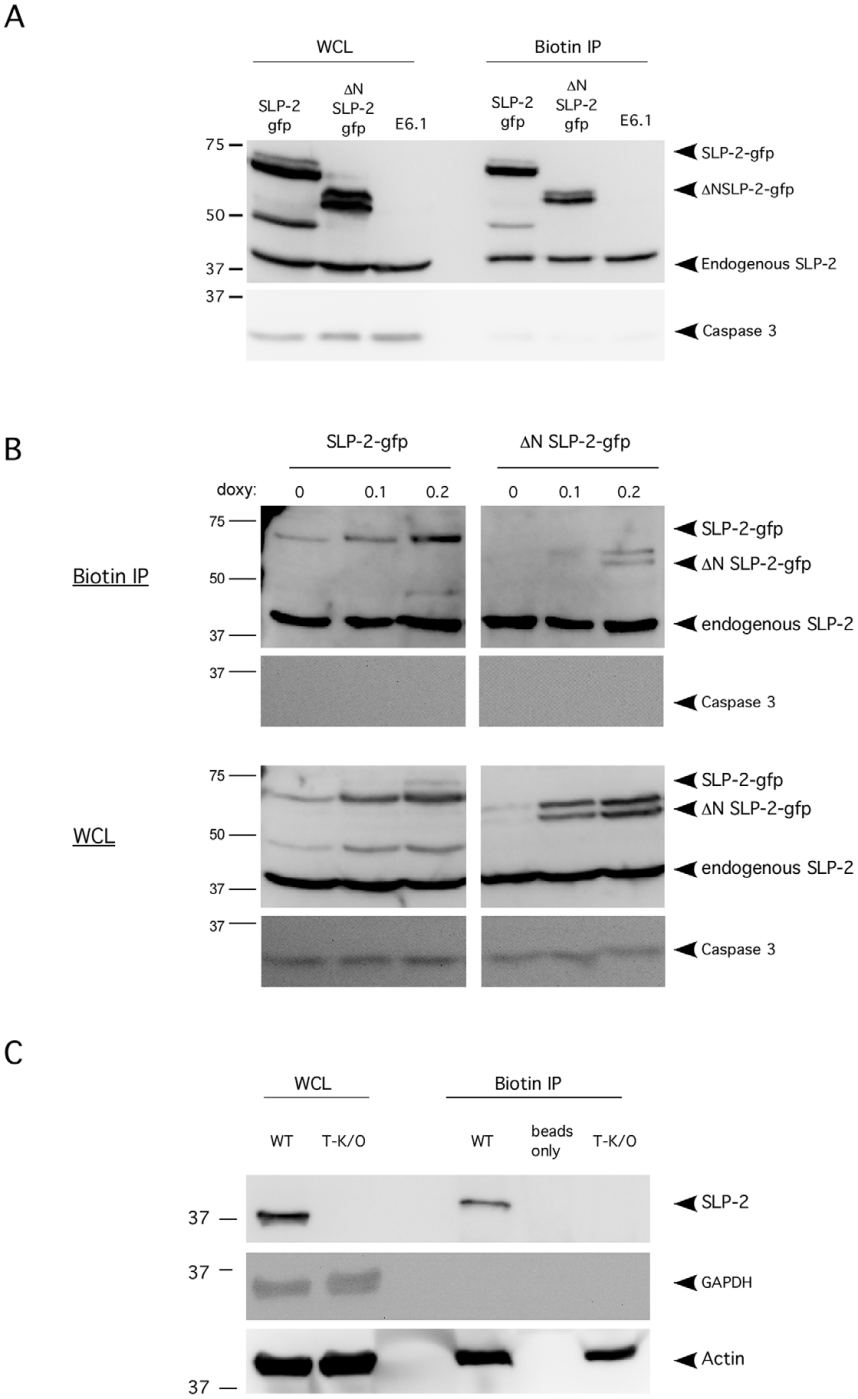
A small pool of SLP-2 is associated with the plasma membrane. A) Intact parental Jurkat T cells, as well as Jurkat T cells stably transfected with full length SLP-2-gfp or ΔN-SLP-2-gfp were biotinylated. Next, cells were lysed and surface proteins were immunoprecipitated with an antibody against biotin. The immunoprecipitate samples (Biotin ip) were immunoblotted for SLP-2 to detect the pool of SLP-2 associated with surface molecules. Whole cell lysates (WCL) from the transfectants were blotted to show expression levels of the transgenes. Samples were also blotted for caspase 3 as a negative control for surface protein pull-down. B) Increasing levels of SLP-2-gfp or ΔN-SLP-2-gfp were induced with increasing concentrations of doxycycline and biotin immunoprecipitation was performed. Whole cell lysates (WCL) and biotin immunoprecipitates (biotin ip) were blotted as in A. C) T cells isolated from wild type and SLP-2 T-K/O mice were analyzed by biotin immunoprecipitation and blotted as in A. Samples were also blotted for actin as a loading control for SLP-2 T-K/O samples and also for GAPDH as a negative control for surface protein pull-down. These results are representative of at least 3 independent experiments.

Although initial studies of SLP-2 have focused on the mitochondrial localization [5], [6], [8], we originally identified SLP-2 in total glycolipid-enriched, detergent-insoluble microdomains from activated T cells [12]. This suggested to us that there may be SLP-2 associated with other membranes, potentially plasma membranes. In order to test the presence of SLP-2 associated with the plasma membrane, T cells were biotinylated and a a biotin immunoprecipitation performed. In these experiments, untransfected Jurkat T cells as well as SLP-2-gfp and ΔN-SLP-2-gfp stably transfected Jurkat T cells were used to determine the association of full length SLP-2 and SLP-2 lacking the mitochondrial targeting domain with T cell surface molecules (figure 2A). Both the endogenous and SLP-2-gfp were found in association with cell surface molecules. In addition, the ΔN-SLP-2-gfp was also found in association with the plasma membrane, indicating that the mitochondrial targeting sequence is not required for association with surface molecules. However, the interaction of ΔN-SLP-2-gfp with membrane proteins was much less efficient because when expression of full length SLP-2-gfp and ΔN-SLP-2-gfp were titrated the full-length SLP-2 associated with the plasma membrane at lower levels of expression compared to ΔN-SLP-2-gfp (figure 2B). Even the very low level of leaky expression of SLP-2-gfp in uninduced cells was sufficient for some SLP-2-gfp to interact with plasma membrane proteins while ΔN-SLP-2-gfp surface pool was only detected when cells expressed high levels of this mutant (by induction with 0.2 µg/mL of doxycycline or higher).

The small surface-associated pool of SLP-2 was also found in mouse T cells. Biotin immunoprecipitation was performed with wild type and SLP-2 T-K/O T cells and SLP-2 was found to co-precipitate with surface proteins in the wild type T cells but not in cells lacking SLP-2 (figure 2C). This result confirms the existence of a plasma membrane-associated pool of SLP-2 in association with surface proteins with an extracellular domain (i.e. biotinylated).

### Homo-oligomerization of SLP-2

**Figure 3:**
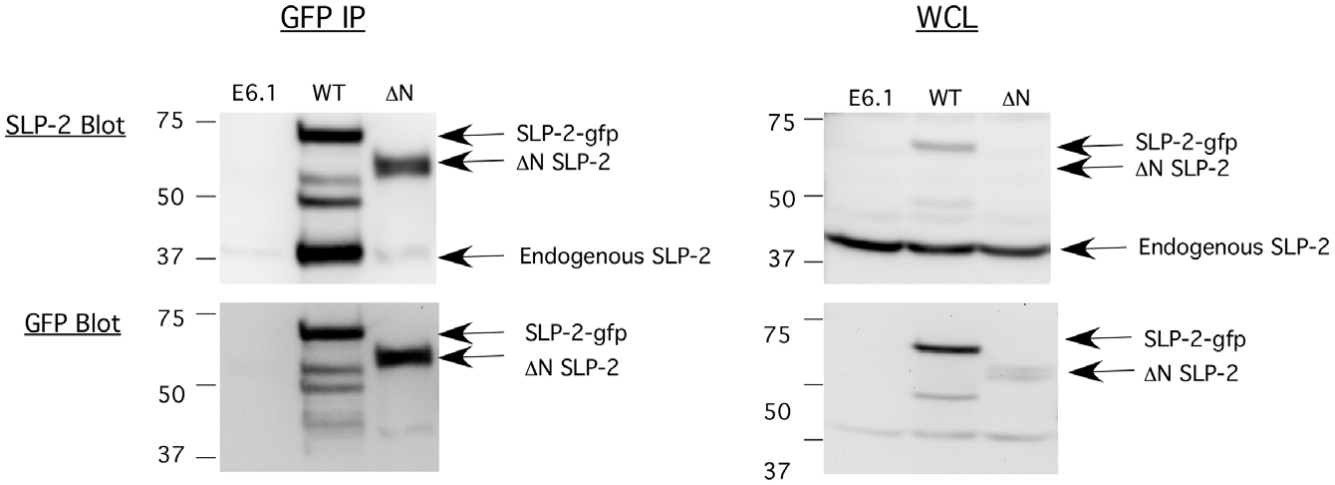
Homo-oligomerization of SLP-2. Parental Jurkat T cells and Jurkat T cells stably transfected with SLP-2-gfp or ΔN-SLP-2-gfp were lysed. Lysates were used for immunoprecipitation with anti-GFP antibodies (GFP ip), and immunoblotted serially for SLP-2 and gfp. Whole cell lysates (WCL) were also blotted to show expression levels of endogenous SLP-2 and the SLP-2 transgenes. This result is representative of at least 3 independent experiments.

We have previously shown that SLP-2 is capable of interacting with numerous proteins, including multiple components of the TCR signalosome and prohibitins [6], [12]. However, it is currently unclear if SLP-2 is capable of homo-oligomerization *in vivo*, without exogenous cross-linkers. This may be an important feature of SLP-2 required to organize specialized membrane microdomains. To test for homo-oligomerization of SLP-2, we utilized the gfp-tagged constructs to examine co-precipitation of endogenous SLP-2 with gfp-tagged SLP-2. In these experiments, parental Jurkat cells, SLP-2-gfp or ΔN-SLP-2-gfp expressing Jurkat cells were immunoprecipitated with anti-gfp antibodies and samples were blotted sequentially for SLP-2 and gfp (figure 3). As expected, gfp immunoprecipitation of the parental cells failed to bring down any proteins, demonstrating the specificity of this system. GFP-immunoprecipitation of SLP-2-gfp co-precipitated endogenous SLP-2, indicating that both are either directly homo-oligomerizing or, alternatively, that they are indirectly associated in larger multi-protein complex. Given the ability of other SPFH family members to homo-oligomerize [15], [16], we favour the first possibility that SLP-2 is capable of homo-oligomerization. Of interest, ΔN-SLP-2-gfp did not co-precipitate with endogenous SLP-2 in significant amounts. This suggests that the N-terminal region of SLP-2, by itself or through its effect on mitochondrial targeting, is important for effective homo-oligomerization of SLP-2.

### SLP-2 Redistributes to the Immunological Synapse During T Cell Activation

**Figure 4:**
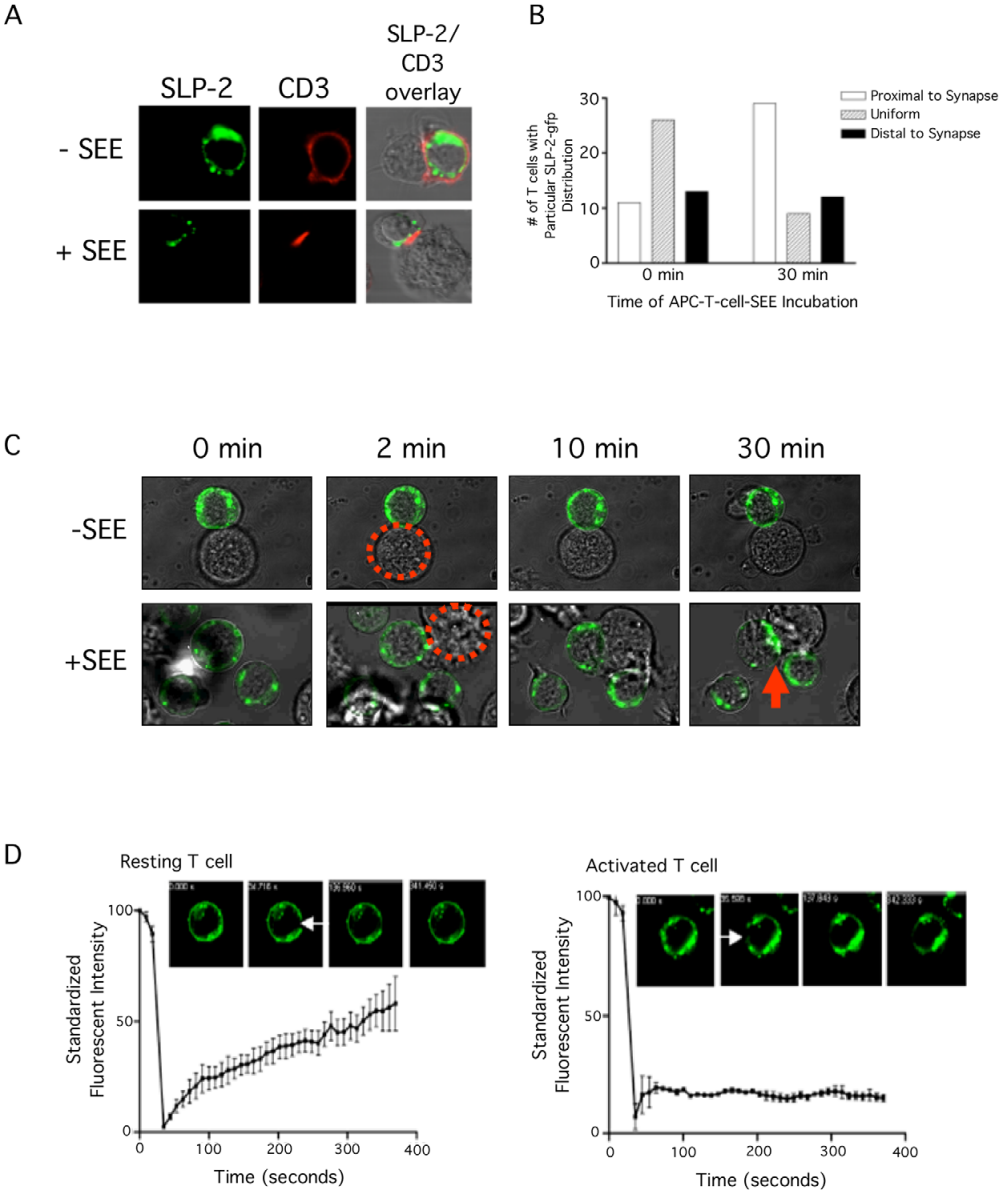
SLP-2 polarizes to the immunological synapse during T cell activation. A) Stable SLP-2-gfp-transfected E6.1 Jurkat T cells were cultured with APC in the presence (+SEE) or absence (−SEE) of SEE and examined by confocal microscopy for the formation of putative synapses (identified as CD3-positive red clusters at the interface between T cell and APC) and for the location of SLP-2-gfp signal (green). Images are representative of at least 50 putative IS. Concomitant studies done with control transfected T cells demonstrated that expression of SLP-2-gfp did not interfere with IS formation. B) Confocal images collected from 50 putative IS images (in A) were analyzed for SLP-2-gfp intracellular localization. SLP-2-gfp signal in un-stimulated (0 min) or stimulated cells (30 min) was classified as predominantly proximal to the IS (white bars), predominantly distal to the IS (black bars), or diffuse throughout the cell (grey bars). C) Redistribution of SLP-2 during T cell activation is shown by a series of videomicroscopy capture images during IS formation. The dotted circle outlines the APC interacting with the Jurkat T cell and the arrow indicates the mature IS with the SLP-2 localization. See Video S1. D) SLP-2-gfp-transfected Jurkat T cells were stimulated with antibodies against CD3 and examined by confocal microscopy. Photobleaching was induced at the arrow-indicated site and regaining of the signal at that site was monitored for 6 minutes at 20 second intervals. Non-specific distribution is documented by progressive regaining of signal with non-bleached SLP-2, while active distribution correlates with lack of regaining of signal. See Videos S2 and S3 for dynamic data.

Next, we investigated the intracellular movement of SLP-2 during T cell activation. Upon activation of SLP-2-gfp stably transfected T cells with APC and SEE, both the plasma membrane-associated pool and the intracellular pool of SLP-2 polarized towards putative immunological synapses, coalescing into major clusters close to the plasma membrane, in the periphery of the IS and underneath the T cell-APC interface (figure 4A). At the cell population level, such polarization was apparent in 60% of T cells forming putative immunological synapses (figure 4B). Video capture of the redistribution of SLP-2-gfp during T cell activation showed that as activation proceeded, greater than 80% of the polarized SLP-2-gfp partitioned in the periphery of the synapse and underneath the putative IS at late time points (figure 4C and Video S1). Only a small fraction of SLP-2 was detectable in the centre of the synapse. Accumulation of SLP-2 in the periphery of the IS mirrored the distribution of pSMACs, known to be enriched in LFA-1. The SLP-2 clusters during T cell activation were actively retained and stable as shown by photobleaching experiments (Figure 4D and Videos S2 and S3). In these experiments, the SLP-2-gfp signal was restored after 400 seconds of bleaching the plasma membrane of non-stimulated T cells but was not restored when SLP-2-gfp clusters in OKT3-stimulated T cells were bleached within the same timeline, indicating that T cell activation was associated with stable SLP-2-gfp clusters unable to freely move within the cell.

### The SLP-2 Pools Predominantly Partition in the p-SMAC of the Immunological Synapse

**Figure 5:**
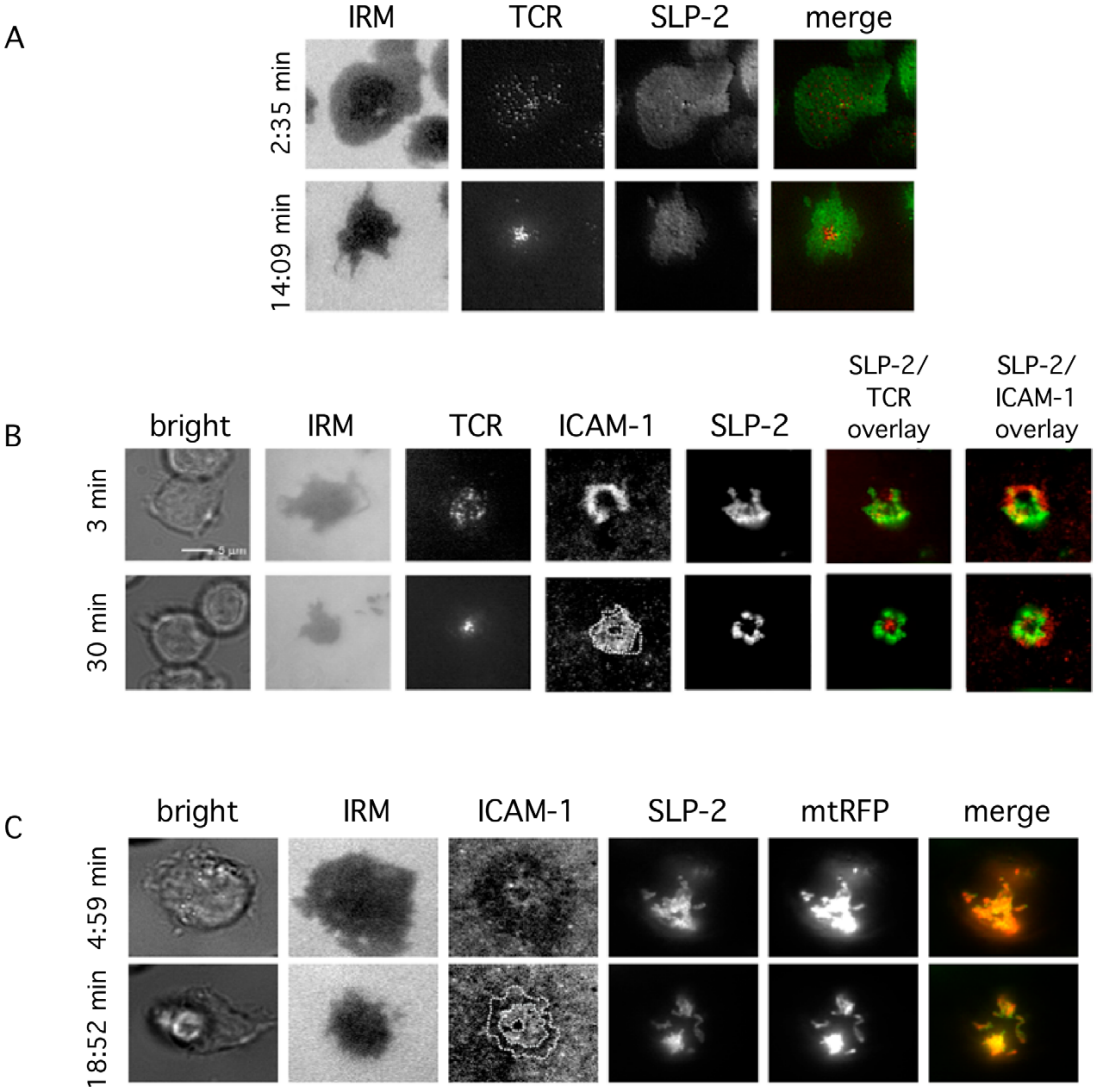
Redistribution of plasma membrane-associated SLP-2 and mitochondria-associated SLP-2 during immunological synapse formation. Jurkat T cells stably expressing SLP-2-gfp were incubated on planar membranes containing ICAM-1 and anti-CD3 to induce immunosynapse formation. Cells were imaged at early (<5 min) and late (15–30 min) stages of immunosynapse formation to demonstrate SLP-2 redistribution upon TCR ligation. A) Plasma membrane-associated SLP-2 redistribution during immunosynapse formation was imaged by TIRFM, to eliminate signal from the mitochondrial pool of SLP-2. Cell contact with the planar membrane was imaged by IRM, TCR images were obtained by wide-field fluorescence microscopy and the image overlay represents SLP-2-gfp as green and TCR as red. B) Total SLP-2-gfp, including both plasma membrane-associated and mitochondrial pools was imaged at early and late stages of immunosynapse formation. The bright field image shows cells being imaged, IRM images show contact with the bilayer as dark areas, and the TCR, ICAM-1 and SLP-2-gfp fluorescence channels are shown in gray scale and two red-green merges (green is always SLP-2). The dotted lines in the ICAM-1 picture represent, from the periphery to the centre of the picture, the outer boundaries of the distal SMAC, of the pSMAC, and of the cSMAC. Images are representative 3 separate experiments. C) SLP-2-gfp expressing Jurkat cells were transfected with mitochondria-targeted RFP to verify mitochondrial localization of intracellular SLP-2-gfp. Images were obtained by wide-field fluorescence microscopy and are representative of three separate experiments. The dotted lines in the ICAM-1 image are as described in B. The percentage of cells showing segregation of the mitochondrial pool of SLP-2 away from the cSMAC is shown in table 1 and this organization of SLP-2 into the pSMAC requires anti-CD3 ligation (table 2).

To further refine our data concerning SLP-2-gfp redistribution to the IS, we used supported planar bilayers containing anti-CD3 and ICAM-1 to which T cells form IS-like structures. This system has excellent optics for resolution of components in the IS. With this system, we visualized the formation of IS by Jurkat cells expressing SLP-2-gfp (figure 5). Under conditions of doxycycline-induced SLP-2-gfp overexpression, we confirmed the presence of two pools of this molecule. The plasma membrane-associated pool of SLP-2 was visualized by total internal reflection fluorescence microscopy (TIRFM) as clusters located at less than 200 nm from the glass plane (i.e., sites of TCR and LFA-1 engagement) (figure 5A). The small plasma membrane pool of SLP-2 distributed uniformly in a granular appearance interspersed with TCR microclusters in the nascent IS (figure 5A). As activation proceeded, the TCR microclusters were surrounded by densely packed SLP-2 clusters. These TCR microclusters weakly excluded the SLP-2 clusters resulting in up to 50% reduction of SLP-2 fluorescence in the cSMACs.

**Table 1:** Segregation of mitochondrial SLP-2-gfp from the cSMAC in the mature IS.

Segregation	No Segregation
25/29 (86.2%)	4/29 (13.8%)

Only cells forming contacts for 3 or more data points by wide field fluorescence microscopy were considered. Images of each field were taken at 2–3 minute intervals. Data is representative of 3 independent experiments.

**Table 2:** Percentage of Jurkat T cells on supported planar bilayers organizing SLP-2-gfp close to pSMAC at the contact interface.

Stimulation	Peripheral ring	No peripheral ring
10 µg/mL OKT3+300 mol/µm^2^ ICAM-1	29^1^	8
300 mol/µm^2^ ICAM-1 only	0	29

1 *P*<0.0001 vs. ICAM-1 only.

In addition, the major mitochondria-associated pool of SLP-2-gfp located at more than 200 nm from the plasma membrane, and also polarized to the IS and segregated from the cSMACs to distribute close to the pSMACs as activation proceeded (figure 5B and Table 1). The mitochondrial origin of this pool of SLP-2 was confirmed by co-localization of the gfp signal with a mitochondria-targeted red fluorescence protein (mtRFP) (figure 5C).

The polarization and partitioning of SLP-2 to the synapse required TCR engagement. In experiments using planar membranes containing only ICAM-1 without anti-CD3, ICAM-1 ligated LFA-1 on the Jurkat T cell to induce immunosynapse formation. Under these conditions, SLP-2 failed to redistribute to the pSMAC and was instead distributed throughout the membrane (Table 2). However, in the presence of both ICAM-1 and anti-CD3 in the planar membrane, immunosynapse formation was accompanied by redistribution of SLP-2-gfp to the pSMAC, with 86% of cells showing exclusion of SLP-2 from the cSMAC (Table 1), indicating the requirement of TCR ligation for SLP-2 redistribution. Together, the results presented here confirm the presence of both a mitochondrial and plasma membrane pool of SLP-2 and demonstrates the redistribution of both SLP-2 pools to surround TCR microclusters in the pSMAC during T cell activation. These data support a general model of SLP-2 function involving the organization of membranes into functional microdomains to support multiple cell functions and specifically T cell activation, as shown here.

### Mitochondrial Recruitment to the IS does not Require SLP-2

**Figure 6:**
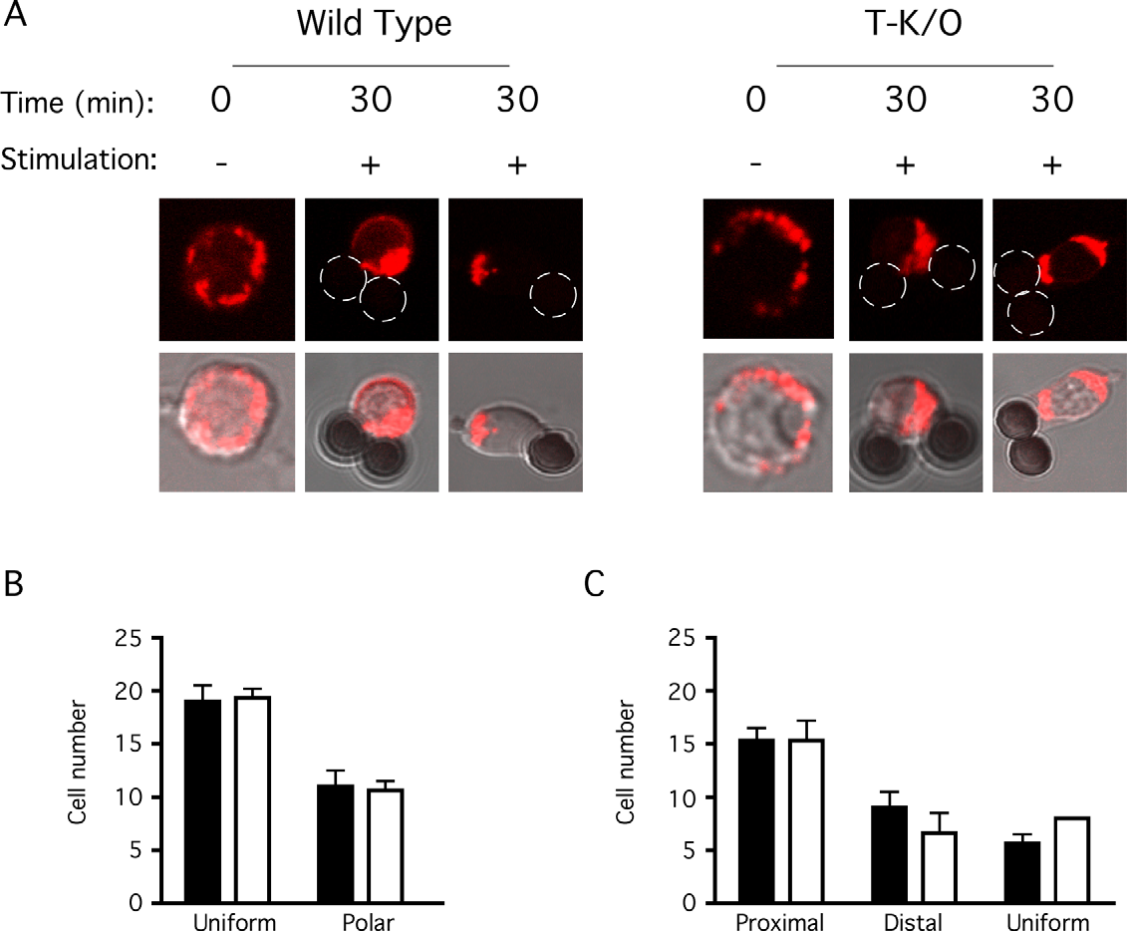
SLP-2-deficient T cells show normal mitochondrial recruitment upon T cell stimulation. T cells were isolated from wild type and SLP-2 T-K/O (T-K/O) mice and stained with MitoTracker Red. Stained cells were plated on poly-L-lysine coated confocal dishes and incubated for 10 minutes to promote adherence. Cells were stimulated for 30 minutes with anti-CD3 and anti-CD28 antibody coated beads or left unstimulated. Cells were then fixed and imaged by confocal microscopy. These images are representative of at least 100 individual cells, imaged in three independent experiments. A) Representative confocal images are shown for wild type and SLP-2-deficient T cells in the absence (0 min) and presence (30 min) of T cell stimulation (2 different cells for each). Mitochondria are shown in red and stimulating beads can be seen in the light image overlay. B) The location of the mitochondria was quantified in un-stimulated wild type (black bars) and SLP-2-deficient (white bars) T cells was quantified as either a polar distribution with mitochondria being clustered together at one end of the cell or a uniform distribution, with mitochondria located throughout the entire cell. C) The location of the mitochondria in stimulated wild type and SLP-2-deficient T cells was quantified as proximal to the stimulating bead, distal to the bead or uniformly distributed throughout the cytoplasm, in a manner similar to that in figure 4. These plots represent an average of 3 independent experiments, in which 30 cells for each group were counted.

It has been shown previously that mitochondria relocate to the IS upon T cell stimulation and that this migration is important for T cell activation [17]. We have previously shown that SLP-2 interacts with the actin cytoskeleton and treatment of Jurkat cells with cytochalasin D inhibits T cell responses [12]. Interestingly, the migration of mitochondria to the IS during T cell stimulation is dependent on microtubules as well as the actin cytoskeleton, and inhibition of actin polymerization inhibits mitochondrial movement and T cell activation [17]. To determine if SLP-2 was involved in mitochondrial translocation to the IS, we imaged mitochondrial movement in response to TCR stimulation in wild type and SLP-2-deficient primary T cells using confocal microscopy (figure 6A). For these experiments, we used cells from conditional SLP-2 knockout mice we have recently generated (Christie *et al*, manuscript submitted), and stimulated these T cells with anti-CD3+ anti-CD28 antibody coated beads for 30 minutes at 37°C [18]. We found that, in unstimulated cells, the mitochondrial population was located primarily around the large nucleus (representative confocal images shown in figure 6A, quantification of mitochondrial localization shown in figure 6B). Upon T cell stimulation, the mitochondrial population re-distributed and was generally found proximal to the stimulating bead, under the IS (figure 5C). In addition, many cells also showed mitochondrial movement to the uropod, classified here as distal localization (figure 6A and 6C), which is characteristic of migrating lymphocytes [19]. Importantly, there was no apparent defect in mitochondrial migration in T cells lacking SLP-2, indicating that mitochondrial movement in T cells is independent of SLP-2.

## Discussion

SLP-2 is a member of the highly conserved stomatin family as well as the SPFH superfamily, consisting of stomatins, prohibitins, flotillins and HflC/K proteins [9]. Although the function of the stomatin family is currently unknown, these proteins are found in glycolipid-enriched, detergent-insoluble microdomains and may be involved in the interaction between protein and lipids in cellular membranes [20], [21], [22]. Specifically, SLP-2 is a mostly mitochondrial protein that has been linked to the formation of cardiolipin-enriched microdomains, thus regulating mitochondrial functions, including energy production and apoptotic responses [6]. SLP-2 has also been identified in detergent-resistant, glycolipid-enriched microdomains of activated T cells and shown to regulate T cell activation through the TCR. The mechanism of action by which SLP-2 does it is unknown [12]. The work presented here investigates the link between T cell activation and the role of SLP-2 in membrane organization. We show that there are two intracellular pools of SLP-2, one major pool associated to mitochondria and another much less abundant pool associated to the plasma membrane. Both pools coalesce at the IS upon TCR ligation, and partition in the peripheral areas of the synapse and are actively maintained in this location during activation. Together, these results suggest that SLP-2 regulates T cell activation by organizing functional microdomains in mitochondrial and plasma membranes. These microdomains can then facilitate the optimal assembly and function of multi-chain complexes or receptors.

The compartmentalization of cellular membranes into specialized microdomains is thought to facilitate the assembly of multi-molecular complexes. This role has been shown in the mitochondrial inner membrane for individual complexes of the respiratory chain and supercomplexes of these individual units, together leading to increased electron transport efficiency and increased ATP production. This role correlates with SLP-2 directly binding to cardiolipin and interacting with prohibitins [6], [12]. Other SPFH family members, such as prohibitin and flotillin are also proposed to organize membrane domains [10], [11]. Alternatively, SLP-2-deficient T cells have less cardiolipin-enriched domains and decreased mitochondrial respiration (Christie *et al*., manuscript submitted), and loss of transmembrane potential [5], [23]. Altogether, the net effect of the increase in SLP-2 levels upon T cell activation would be an increase in cardiolipin-enriched microdomains and enhanced mitochondrial function resulting in increased T cell function.

The data presented here extends the role of SLP-2 as an organizer of membrane compartmentalization to the plasma membrane. Upon TCR ligation, there is recruitment of a large number of adapter and signalling molecules to form the TCR signalosome [24]. Assembly of this multi-molecular complex is also associated with re-organization of plasma membranes, through the aggregation of detergent-resistant, glycolipid-enriched microdomains previously referred to as lipid rafts [25], [26], [27], [28], and the formation of an IS [29]. Within the IS, stable signalling TCR microclusters containing kinases and adapters are detected in the outer ring of the synapse together with LFA-1 and other adhesion molecules forming the pSMAC [30]. As activation proceeds, TCR microclusters move from the periphery to the centre of the synapse (cSMACs), destined for internalization and degradation [31], [32], [33], a process that ensures signal down-regulation [34]. The results presented here are compatible with the idea that SLP-2 facilitates the assembly of the TCR signalosome during T cell activation. In a manner similar to Mec-2 and podocin, members of the SPFH superfamily, SLP-2 may associate with cholesterol at the plasma membrane and with components of the TCR signalosome the stability of this multimolecular complex [7], [12]. The ability of SLP-2 to homo-oligomerize may allow SLP-2 to form a ring-like structure around the signalosome to separate components from other proteins at the plasma membrane and to prevent dissociation of the complex, in a manner similar to what SLP-2:prohibitin complexes may do in mitochondria [6], [11]. A detailed characterization of the TCR signalosome under conditions of SLP-2 deficiency is currently underway as part of the analysis of the conditional, T cell-specific SLP-2 knockout mouse (Christie et al, manuscript submitted).

How SLP-2 specifically interacts with cardiolipin in the mitochondrial membrane and other lipids in the plasma membrane is an issue for further investigation. SLP-2 lacks a putative transmembrane domain and palmitoylation sites found in other stomatin family members [5]. We have previously indicated that there may be putative myristoylation sites at the amino-terminus of SLP-2, which may facilitate membrane association [12]. Alternatively, the SPFH domain may be directly involved in this association as it has been shown for the superfamily members Mec-2 and podocin and its binding to cholesterol and for SLP-2 and its binding to cardiolipin [6], [7].

Using video capture microscopy we have shown that the mitochondrial-associated pool of SLP-2 redistributed to the IS upon T cell activation. This result corroborates the observation of mitochondrial relocation to the IS upon T cell stimulation [17]. In this work, the authors suggest a model in which mitochondria act as a localized calcium sink to maintain Calcium Release Activated Calcium (CRAC) channel activation, strengthening T cell signalling and activation. Studies looking at knockdown of SLP-2 with siRNA have shown that the loss of SLP-2 leads to decreased calcium uptake by the mitochondria [13]. Similarly, we have found that mitochondrial calcium uptake is increased in cells over-expressing full-length SLP-2 (C. Lemke and J. Madrenas, unpublished observations). However, it is currently unclear how SLP-2 modulates calcium uptake by the mitochondria. While an inhibitor of mitochondrial sodium/calcium exchange channel equalized the rate of calcium extrusion from the mitochondria in control and SLP-2-depleted cells, it did not restore full calcium uptake into the mitochondria [13], indicating that calcium exchange rates alone cannot account for the role of SLP-2 in mitochondrial calcium uptake.

Since we had previously shown that SLP-2 interacts with actin and it has been shown that the mitochondrial translocation to the IS requires actin polymerization [12], [17], it was plausible that SLP-2 may link the mitochondria to the actin cytoskeleton and facilitate the movement of mitochondria to the IS. However, our data indicate that this is not the case because we found that mitochondrial translocation to the IS proceeded normally in SLP-2-deficient T cells. Thus, SLP-2 is not required for mitochondrial translocation to the IS during T cell activation.

The identification of SLP-2 and other mitochondrial proteins at the plasma membrane may reflect membrane exchange between these organelles and other cellular compartments and organelles, and point to a novel pathway of intracellular trafficking. We have confirmed the presence of a mitochondrial targeting sequence in the amino-terminus of SLP-2. This raised the question of the intracellular trafficking pathway leading to the presence of SLP-2 in the cell surface. It is possible that the plasma membrane pool of SLP-2 arises by default when a small proportion of newly synthesized SLP-2 fails to be transported to the mitochondria. However, it is also possible that a small amount of membrane exchange occurs between the mitochondrial and plasma membranes, allowing for the exchange of membrane-associated proteins, which is supported by the existence of mitochondria-derived vesicles and its fusion with other organelles such as lysosomes [35], [36], [37], [38]. In addition to SLP-2, a small surface pool has also been shown for other mitochondrial proteins such as prohibitin, porin, NADH dehydrogenase, ubiquinol-cytochrome c reducatase and ATP synthase, indicating that the surface association of mitochondrial proteins is not unique to SLP-2 [39], [40], [41], [42]. The purpose of mitochondrial proteins at the plasma membrane is unclear, although the association of SLP-2 with components of the T cell signalosome suggest that both pools of SLP-2 are likely functional.

In summary, the work presented here provides the first evidence of two pools of SLP-2 in human and mouse T cells and it suggests to a conservation of function at both membranes. In the mitochondria, SLP-2 organizes the membrane into cardiolipin-enriched domains to facilitate respiratory chain function. At the plasma membrane, SLP-2 may facilitate immunosynapse organization, leading to increased T cell signalling and activation. It is likely that both of these functions have the same mechanistic basis, i.e. organization of specialized microdomains that facilitate the assembly of multi-molecular complexes and ultimately ensure their optimal function.

## Materials and Methods

### Cells

Jurkat T cells (E6.1) were obtained from American Type Culture Collection (Manassas, VA) and cultured in supplemented RPMI 1640 medium. The B lymphoblastoid cell line, LG2, was kindly provided by Dr. Eric Long (NIAID, NIH, Rockville, MD) and cultured in standard supplemented RPMI 1640 media.

### Plasmids, siRNA and T Cell Transfectants

Human SLP-2 cDNA was subcloned into the pEGFPN1 expression vector (Clontech Inc. Palo Alto, CA) to create an in-frame translational fusion of the 3′ end of SLP-2 to gfp, as previously described [18], [43]. Subsequently, the SLP-2-gfp fusion was cloned into the doxycycline inducible pBIG2i vector [18]. A mutant construct lacking the amino-terminal region (deletion of amino acid residues 1–77) was generated and cloned into pBIG2i. Stable transfectants were generated by electroporating linearized plasmid into Jurkat E6.1 T cells and screening for stable expression of SLP-2-gfp. Doxycycline (Sigma, St. Louis, MO) was added to overnight cultures at 1 µg/mL to induce SLP-2-gfp expression, which was monitored by direct flow cytometry (Becton Dickinson [BD], San Jose, CA).

### Mice

A full-length genomic fragment containing the mouse SLP-2 gene was cloned within the lox sites of the 3LoxP3NwCD vector. Upon sequence confirmation, the plasmid was electroporated into C57BL/6 embryonic stem cells and clones were selected for neomycin resistance. The resistant clones were screened by Southern blot and PCR analysis to confirm homologous recombination of the SLP-2-floxed DNA sequence. Clones containing the SLP-2-floxed sequence were injected into B6/Try blastocysts and implanted into a pseudo-pregnant mouse. The offspring was selected by chimeric coat colour and backcrossed with C57BL/6 mice to produce SLP-2^lox/wt^ mice in the C57BL/6 background. SLP-2 floxed mice were crossed with C57BL/6 mice transgenic for Cre recombinase under the control of the CD4 promoter, purchased from Taconic (Hudson, NY) [44], to generate a T cell specific SLP-2 knockout strain (SLP-2 T-K/O, Christie *et al*, manuscript submitted). SLP-2 floxed mice lacking Cre were used as control mice. Breeding colonies were derived from the same original SLP-2 floxed breeders and kept in parallel. Mice were maintained in the animal facility at the University of Western Ontario with approval from the Animal Use Subcommittee in accordance with the Canadian Council on Animal Care Guidelines.

### Antibodies

A rabbit polyclonal antiserum against human SLP-2 was generated by immunization with a peptide spanning amino acids 343 to 356 (ProSci Inc., Poway, CA). Polyclonal antibodies against SLP-2 (Protein Tech Group, Chicago, IL) and gfp (BD Bioscience, San Jose, CA) were used in these studies. Pre-immune rabbit serum was used as an isotype control (ProSci Inc., Poway, CA). Monoclonal antibodies against β-actin (Santa Cruz Biotechnology, Santa Cruz, CA), the α-subunit of Complex V (MitoSciences, Eugene, OR), biotin (Jackson ImmunoResearch, West Grove, PA), Caspase 3 (Cell Signaling, Danvers, MA), GAPDH (Chemicon International, Temecula, CA) and PE-labelled anti-CD3 (UCHT-I) were used (BD Bioscience, San Jose, CA) were used.

### Mitochondria Isolation

Intact mitochondria were isolated from 20×10^6^ Jurkat T cells, SLP-2 T-K/O or wild type control cells using the Qproteome Mitochondria Isolation Kit (Qiagen). Cytosolic fractions were concentrated in Amicon Ultra-0.5 centrifugal filter units with Ultracel-30 membrane (Millipore, Billerica, MA). Mitochondrial and cytosolic preparations were mixed with sample buffer containing β-mercaptoethanol, boiled, and analyzed by sodium dodecyl sulfate-polyacrylamide gel electrophoresis (SDS-PAGE).

### Immunoprecipitations

Protein A or protein G agarose beads were coated with either SLP-2 antisera, pre-immune sera, anti-gfp or anti-biotin antibodies overnight at 4°C [43]. Beads were washed to remove excess antibody and cell lysates were incubated with the antibody-coated beads overnight at 4°C. Following immunoprecipitation, unbound proteins were removed and antibody-bound protein complexes were eluted in sample buffer with β-mercaptoethanol. Immunoprecipitated samples were separated by SDS-PAGE and immunoblotted with the appropriate antibodies.

### Cell Surface Biotinylation

Jurkat or mouse T cells were incubated with EZ-link sulfo-NHS-biotin (Pierce, Rockford, IL) in PBS pH 8.0, for 30 minutes at room temperature. Cells were then washed three times with cold PBS, lysed and immunoprecipitated for biotin [18], [45]. Lysates and immunoprecipitates were separated by SDS-PAGE and blotted with antibodies against SLP-2.

### Confocal Microscopy

Confocal microscopy was performed with a Zeiss LSM 510 microscope. Jurkat T cells or murine T cells (1×10^6^/ ml) were incubated on poly-lysine-coated (0.01%, Sigma) glass bottom microwell dishes (MatTek Corp., MA) for 10 minutes to promote cell adherence at 37°C. SLP-2-gfp localization and mitochondrial movement was monitored by staining cells with 100 nM MitoTracker Red CMXRos (Invitrogen, Burlington, ON, Canada) for 15 min at 37°C in complete RPMI 1640. SLP-2-gfp distribution during IS formation was assessed by culturing doxycycline-induced SLP-2-gfp stably transfected T cells with LG2 antigen-presenting cells (APC) pre-incubated with 1 µg/ml staphylococcal enterotoxin E (SEE) for either 10 or 30 minutes. Following the allotted time of co-incubation, the T cell-APC conjugates were rapidly fixed with 4% paraformaldehyde, washed with PBS+1% FCS and stained with PE conjugated anti-CD3 for 30 minutes on ice. For experiments imaging mitochondrial localization in primary mouse cells, T cells were isolated by magnetic separation using the MACS pan T cell isolation kit (Miltenyi Biotec, Auburn, CA). Cells were stained with MitoTracker Red, stimulated with dynabeads mouse T activator CD3/CD28 (Invitrogen, Burlington, ON, Canada) for 30 minutes and cells were fixed and imaged [46]. For experiments using planar membranes, glass-supported dioleoylphosphatidylcholine bilayers incorporating Cy5-ICAM-1 (300 molecules/µm^2^) and 0.1% cap-biotin were prepared in a Bioptechs flow cell. Unlabelled Streptavidin (8 µg/mL) and Cy3-conjugated anti-human CD3 (OKT3 clone, 10 µg/mL), were loaded sequentially in HBS/HSA buffer (Hepes buffered saline supplemented with 5 mM glucose, 2 mM MgCl2, 1 mM CaCl2, and 1% human serum albumin). Jurkat T cells were also suspended in HBS/HAS buffer. All microscopy was performed on an automated microscope with a Hamamatsu USA Orca-ER cooled CCD camera. The hardware on the microscope was controlled using IPLAB software (Scanalytics) on a PowerMac G4 Macintosh computer. Images were exposed in wide-field for 1–2 s at a resolution of 0.11 µm per pixel using the 60×1.45 N.A. objective. Interference reflection microscopy (IRM) is based on destructive interference in green light reflected from the bilayer-cell interface leading to a dark area where cells are in close contact with the bilayer. Images of cell interaction with the planar membrane were collected by IRM and fluorescent images to examine re-localization of T cell proteins were collected by total internal reflection fluorescence microscopy (TIRFM). Images were inspected using Metamorph (Molecular Devices).

## Supporting Information

Video S1 **Localization of SLP-2 during IS formation.** Antigen presenting cells (APCs) were incubated overnight with SEE (1 µg/ml), washed, resuspended in medium, and incubated on polylysine-coated glass bottom microwell dishes for 10 minutes to allow cells to adhere. SLP-2-gfp-expressing Jurkat T cells were added to the APCs preincubated with SEE on the polylysine-coated microwell dishes. Once T:APC doublet was identified, images were acquired every 30 seconds for 60 minutes. The majority of SLP-2-gfp localized proximal to the synapse and distributed evenly in early stages, and as the IS matures SLP-2-gfp moved to periphery of the synapse.
(MOV)

Video S2 **SLP-2 clusters are not static in non-stimulated T cells.** Jurkat T cells stably expressing SLP-2-gfp were incubated on polylysine-coated glass bottom microwell dishes for 10 minutes to allow cells to adhere. Adherent, SLP-2-gfp expressing Jurkat T cells were then placed under confocal microscope. Forty images were acquired over a 375 seconds, with three images acquired before photobleaching. Area of cell indicated by arrow was photobleached using 100% of laser power for 3 seconds and the movement of SLP-2-gfp back into photobleached area was measured. Note that, in non-stimulated cells, there is significant recovery of SLP-2-gfp in photobleached area within the examined time window.
(MOV)

Video S3 **Stability of SLP-2 clusters during T cell activation.** Jurkat T cells stably expressing SLP-2-gfp were incubated on polylysine-coated glass bottom microwell dishes for 10 minutes to allow cells to adhere. Adherent Jurkat T cells were then stimulated with OKT3 (1 µg/ml) for 15 minutes and then placed under confocal microscope. Forty images were acquired over a 375 seconds, with three images acquired before photobleaching. Area of cell indicated by arrow was photobleached using 100% of laser power for 3 seconds and the movement of SLP-2-gfp back into photobleached area was measured. Note that, in the stimulated T cell, it corresponds to an area with SLP-2-gfp clusters. Stimulated Jurkat T cells show little to no recovery of gfp signal in photobleached area within the examined time window.
(MOV)
